# A ResNet mini architecture for brain age prediction

**DOI:** 10.1038/s41598-024-61915-5

**Published:** 2024-05-16

**Authors:** Xuan Zhang, Si-Yuan Duan, Si-Qi Wang, Yao-Wen Chen, Shi-Xin Lai, Ji-Sheng Zou, Yan Cheng, Ji-Tian Guan, Ren-Hua Wu, Xiao-Lei Zhang

**Affiliations:** 1 College of Engineering, Shantou University, Shantou, 515063 China; 2 College of Computer Science, Sichuan University, Chengdu, 610065 China; 3 Department of Radiology, Second Hospital of Shandong University, Jinan, 250033 China; 4 Department of Radiology, Second Affiliated Hospital of Shantou University Medical College, Shantou, 515041 China

**Keywords:** Brain age prediction, MRI, Deep learning, Lightweight network, ResNet, Computer science, Medical research

## Abstract

The brain presents age-related structural and functional changes in the human life, with different extends between subjects and groups. Brain age prediction can be used to evaluate the development and aging of human brain, as well as providing valuable information for neurodevelopment and disease diagnosis. Many contributions have been made for this purpose, resorting to different machine learning methods. To solve this task and reduce memory resource consumption, we develop a mini architecture of only 10 layers by modifying the deep residual neural network (ResNet), named ResNet mini architecture. To support the ResNet mini architecture in brain age prediction, the brain age dataset (OpenNeuro #ds000228) that consists of 155 study participants (three classes) and the Alzheimer MRI preprocessed dataset that consists of 6400 images (four classes) are employed. We compared the performance of the ResNet mini architecture with other popular networks using the two considered datasets. Experimental results show that the proposed architecture exhibits generality and robustness with high accuracy and less parameter number.

## Introduction

The human brain structure exhibits the age-related changes across the lifespan, which may reveal several risks of encountering health-related issues at different stages of life^1^. Age-related brain changes are associated with the etiology of brain diseases, especially neurodegenerative diseases (Alzheimer’s disease, Parkinson’s disease and amyotrophic lateral sclerosis)^2^. The process of age-associated brain diseases varied greatly across the population, surprisingly neuroimaging such as structural magnetic resonance imaging (MRI) that allows brain tissues with visualizing details and subtle changes can provide a comprehensive solution for the task. Based on the different pathological manifestations of patients of different ages, brain age prediction through neuroimaging can be one of the important aspects of diagnosis.

In this context, neuroimaging-derived models aided by machine learning has been successful in solving different tasks of brain age prediction, mostly using MRI scans. Particularly, deep learning has become prevalent in manifold brain age estimation, allowing advanced ability to learn and represent image features^3^. In a recently reported literature^1^, an overview of brain age prediction and the available tools (deep learning architectures) have been summarized. This paper reviews the publications of brain age estimation using deep learning architectures from neuroimaging data, including convolutional neural network (CNN)^3–10^, ensemble CNNs^11–13^ and Transformer based models^14–17^. Despite the saturation of performance metrics on datasets and the intricate state-of-the-art advancements, it suggests that “computational complexity” is one of research niches that deserve further attention^1^. For this, Fisch et al.^7^ introduced a ResNet-based 2-layer 3D CNN architecture. They employed a preprocessing technique that involves brain image cropping to reduce the computational complexity of the model. However, this approach results in disconnected patches within the slices, leading to the loss of certain contextual features present in the images. In addition, Lam et al.^18^ proposed a 2D recurrent neural network for predicting brain age. The main objective was to reduce the parameter count. In comparison to 3D CNN models, the model had 10,680,605 parameters, which is half that of the 3D CNN model. Ballester et al.^19^ proposed slice-level brain age prediction using a combination of CNNs and linear regression. Their work on brain age prediction was carried out through multivariate analysis, in which ensemble model integrates multiple networks to enhance predictive performance and price usually needs higher complexity of training. The challenges encountered are similar to those faced by other researchers, where reducing computational complexity becomes imperative while ensuring accuracy. Herein, we seek to break through this barrier. While the challenge of computational complexity is well-acknowledged, our novel contribution in this paper is the introduction of a lightweight network, which tackles imbalance in both efficiency and accuracy.

Our main contributions can be summarized as follows. To be effective, neural networks for brain age prediction should be lightweight, in which the better spatial inductive biases allow networks to learn representations with fewer parameters. The more flexible deployment behaved by lightweight network would reduce the costs of using infrastructure and accelerate response speed. Towards this end, we develop a network with only 10 layers by modifying deep residual neural network (ResNet)^20^, named as ResNet mini (ResMini) architecture^21^, to extract the features of considered datasets. Case studies indicate that the proposed network exhibits competitive results when we comprehensively consider network accuracy and parameter number.

The rest of this paper is structured as follows. In section “Results”, we assess our ResMini by comparing it with ResNet18^20^, BHCnet^22^. We conclude our research in section “Discussion” and suggest avenues for future research. Section “Materials and methods” introduces the used datasets, and provide a detailed description of our proposed approach to address the challenge of computational complexity by ResMini.

## Results

**Figure 1:**
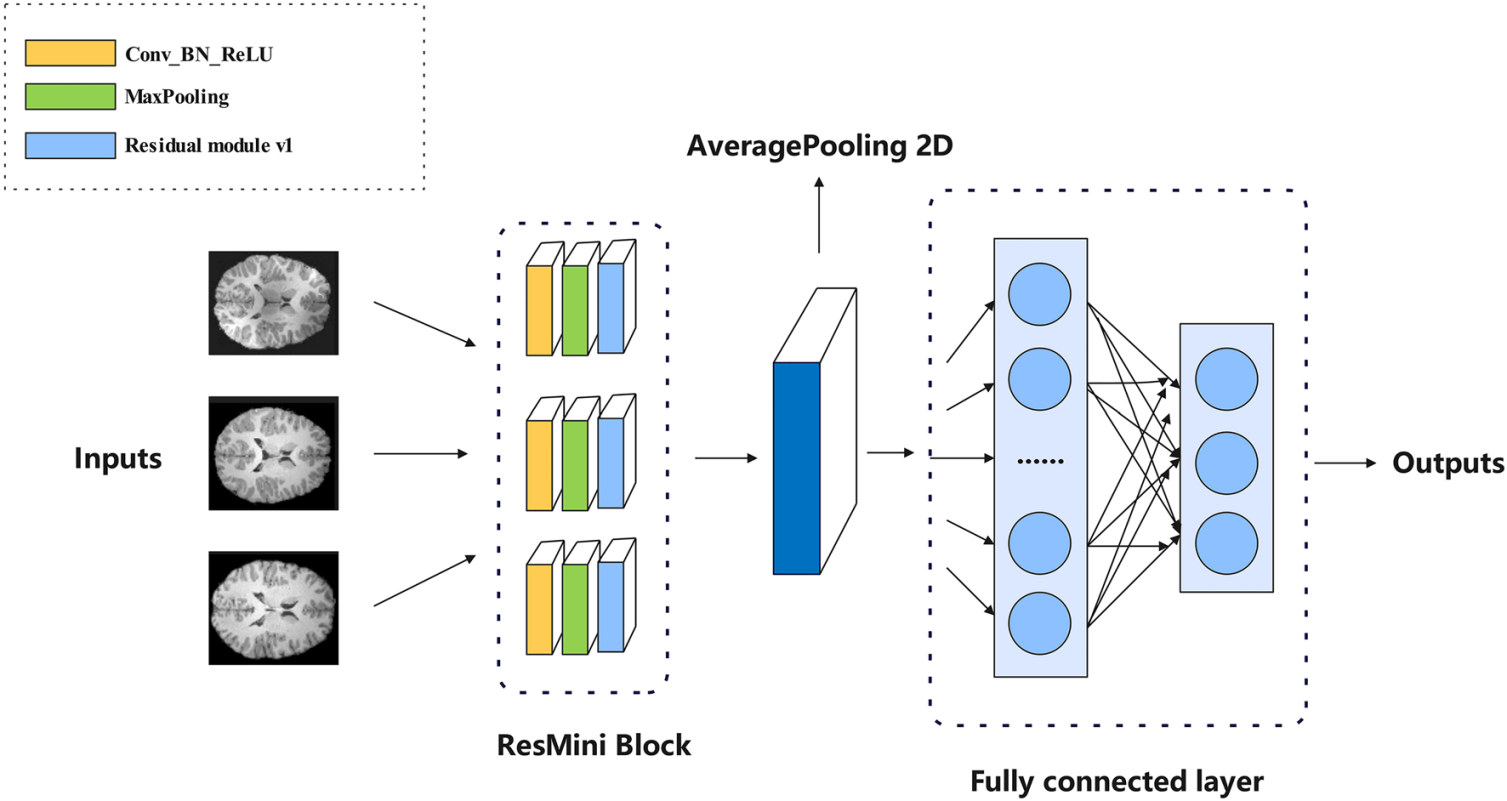
Overview of the ResMini based brain age prediction framework.

To tackle the study task, the ResMini based method is described in Fig. 1. To evaluate the advantages and disadvantages of our model in brain age prediction, we first use ResNet18^20^ and BHCnet^22^ to replace our ResMini block in the same experimental environment using the brain age dataset (OpenNeuro #ds000228) to achieve classification outcomes in three classes (https://openneuro.org/datasets/ds000228/versions/1.1.0). To validate the generality and robustness of the ResMini, the Alzheimer MRI preprocessed dataset that consists of 6400 images (four classes) is also considered (https://www.kaggle.com/datasets/sachinkumar413/alzheimer-mri-dataset).

**Figure 2:**
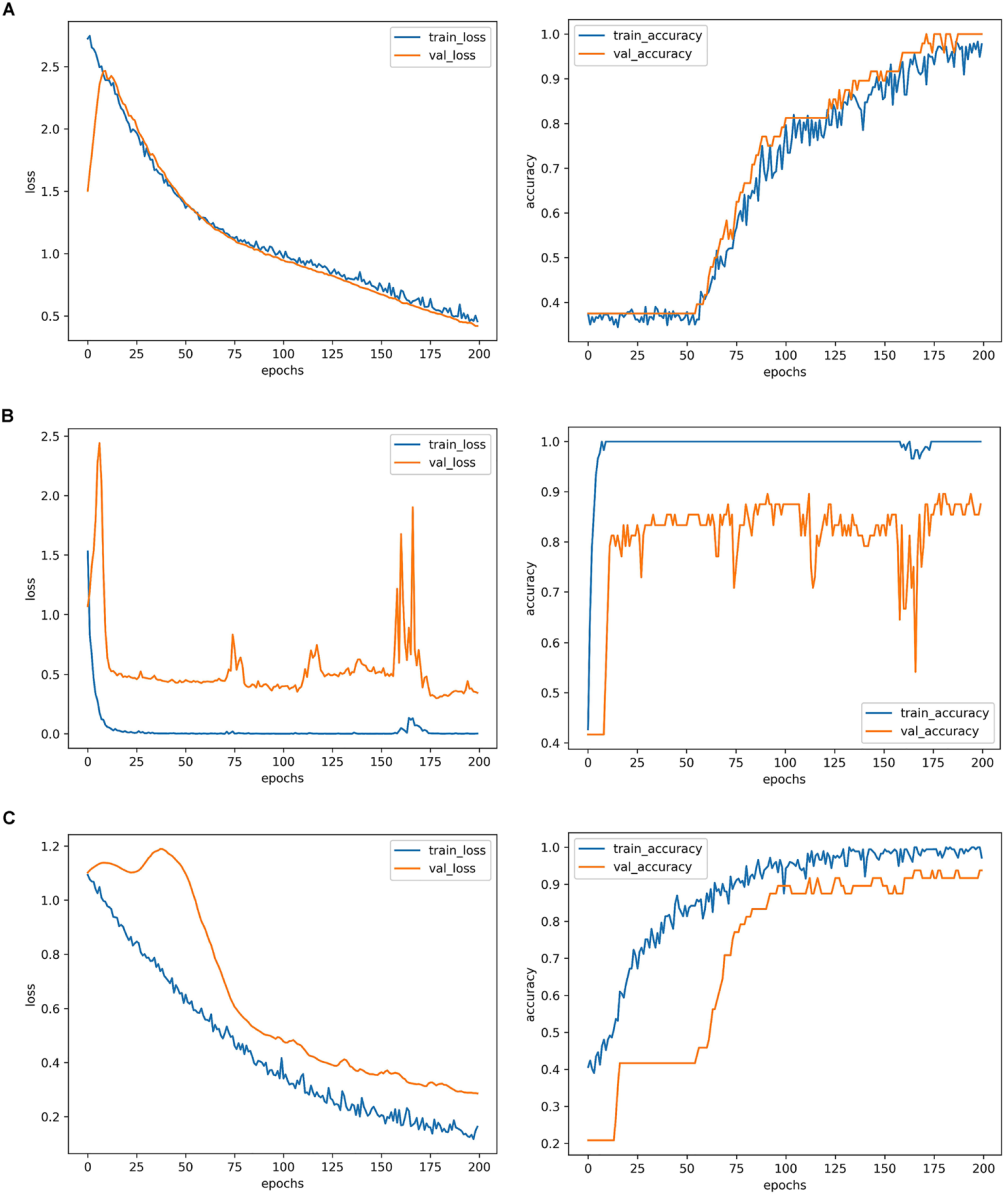
The loss function curves and accuracy curves of the three networks when they are applied to the brain age dataset (OpenNeuro #ds000228). (**A**) BHCNet results, (**B**) ResNet18 results, (**C**) ResMini results.

Figure 2 shows the loss and accuracy curves of the three networks when they are applied to the brain age dataset. From the left column of Fig. 2, we find that the iterations of BHCnet and ResMini are more than that of ResNet18 in training loss, where the needed iteration of ResNet18 is only 25 iterations when the training loss approaches to the stability. However, there is high level of fluctuating in the validation loss of ResNet18. What is more, the terminal value of the validation loss obtained by ResMini is lower than those of BHCnet and ResNet18. At the same time, we compare the accuracy curves using the three networks, as shown in the right column of Fig. 2. It is found that the accurate convergence of training caused by the ResNet18 increases sharply, yet the high level of fluctuating exists in its validation accuracy curve. In contrast, BHCnet and ResMini show steady increasing tendency in the accuracy curves of training and validation.

**Figure 3:**
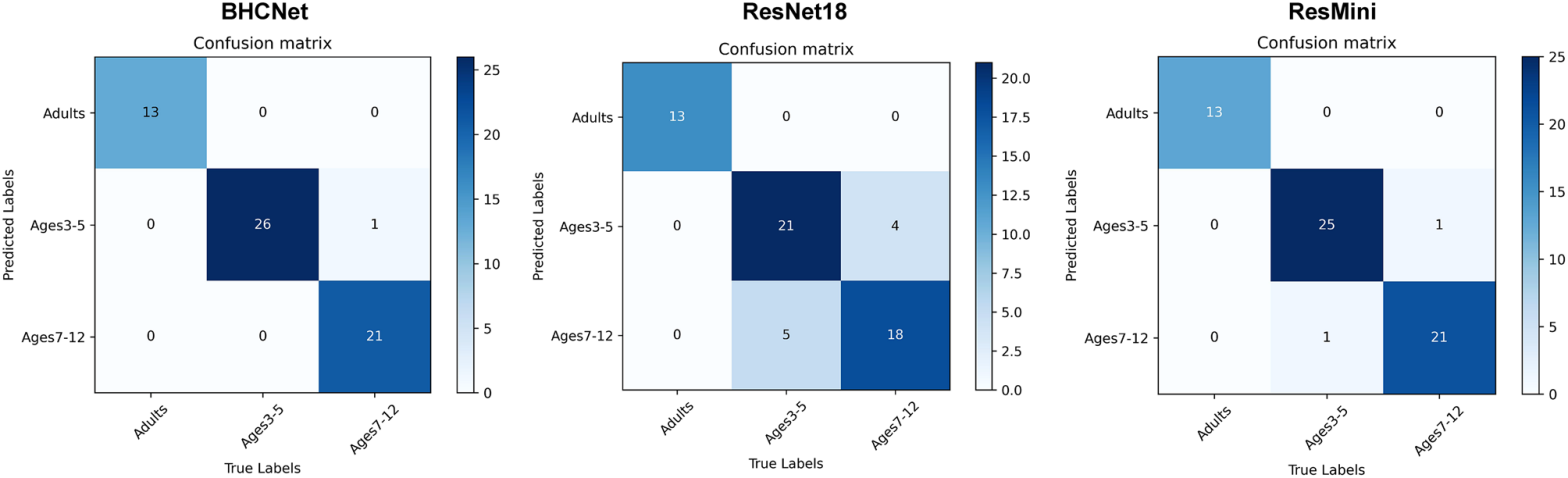
Confusion matrices of the three networks when they are applied to the brain age dataset (OpenNeuro #ds000228).

Figure 3 shows the confusion matrices of the three networks on the brain age dataset. The test set consists of 61 data, including 27 samples of 3–5 years old, 21 samples of 7–12 years old, and 13 samples of adult. By using the ResMini, 1 and 1 are wrongly predicted for 3–5 years old and 7–12 years old respectively, and 13 data is correctly predicted among 13 samples of adult. In BHCNet prediction, only 1 is wrongly predicted for 7–12 years old. It is found that 5 and 4 are wrongly predicted for 3–5 years old and 7–12 years old in ResNet18 prediction, respectively.

Table 1 summarizes the comparison results in terms of accuracy and efficiency, as well as the number of parameters, when ResMini, BHCnet and ResNet18 are applied to the test set for brain age prediction. The results show that the performance of the ResMini is almost same as that of the BHCnet and slightly exceeds ResNet18 in terms of accuracy, while it outperforms the performance of BHCnet and ResNet18 in parameter number. The execution times of 70.25 s, 77.47 s, and 115.89 s are obtained by ResMini, BHCnet and ResNet18, respectively. Therefore, ResMini exhibits shorter execution times and higher efficiency. In addition, the results of Table 1 reveal that the parameter number of ResMini are less than those of BHCnet and ResNet18, while the parameter number of ResMini is only 50.31% and 0.88% of its counterparts. Specifically, the prediction accuracy of ResMini for three age group categories are as follows: 100% for adults, 96.1% for 3–5 years old, and 95.4% for 7–12 years old (Table 2). Among these, the accuracy of adults is higher by 3.9% compared to 3–5 years old, which may be attributed to the imbalanced distribution of samples among different classes. Consequently, the model might tend to predict the class with a higher sample count more accurately, leading to discrepancies in accuracy across different classifications.

**Table 1:** Performance of the three networks for the brain age dataset (OpenNeuro #ds000228) when we consider accuracy, parameter number, and overall execution time in test set.

	ResMini	ResNet-18	BHC_net
Accuracy	0.967	0.958	0.970
Total parameters	98,907	11,181,379	196,595
Execution time	70.25 s	77.47 s	115.89 s

**Table 2:** Prediction results of three age group categories when ResMini is applied to the brain age dataset (OpenNeuro #ds000228).

	OpenNeuro dataset		
Accuracy	Adults	1	0.967
	Ages 3–5	0.961	
	Ages 7–12	0.954	
Recall	0.972		
Precision	0.972		

Table 3 illustrates the performance of ResMini before and after data augmentation when it is applied to the brain age dataset. The accuracy of 96.7% is obtained after data augmentation, which exhibits an improvement of 22.6% compared to the pre-augmentation stage. Thus, data augmentation significantly contributes to enhancing the model’s generalization capability. It is noteworthy that the training set’s accuracy surpassed that of the testing set before data augmentation. However, this gap has been mitigated after data augmentation, effectively preventing overfitting.

**Table 3:** Performance of the ResMini for the brain age dataset (OpenNeuro #ds000228) before and after data augmentation.

	Sample number	Train accuracy	Test accuracy
Raw dataset	155	0.917 (± 0.02)	0.741
Dataset after data augmentation	310	0.983 (± 0.02)	0.967

**Figure 4:**
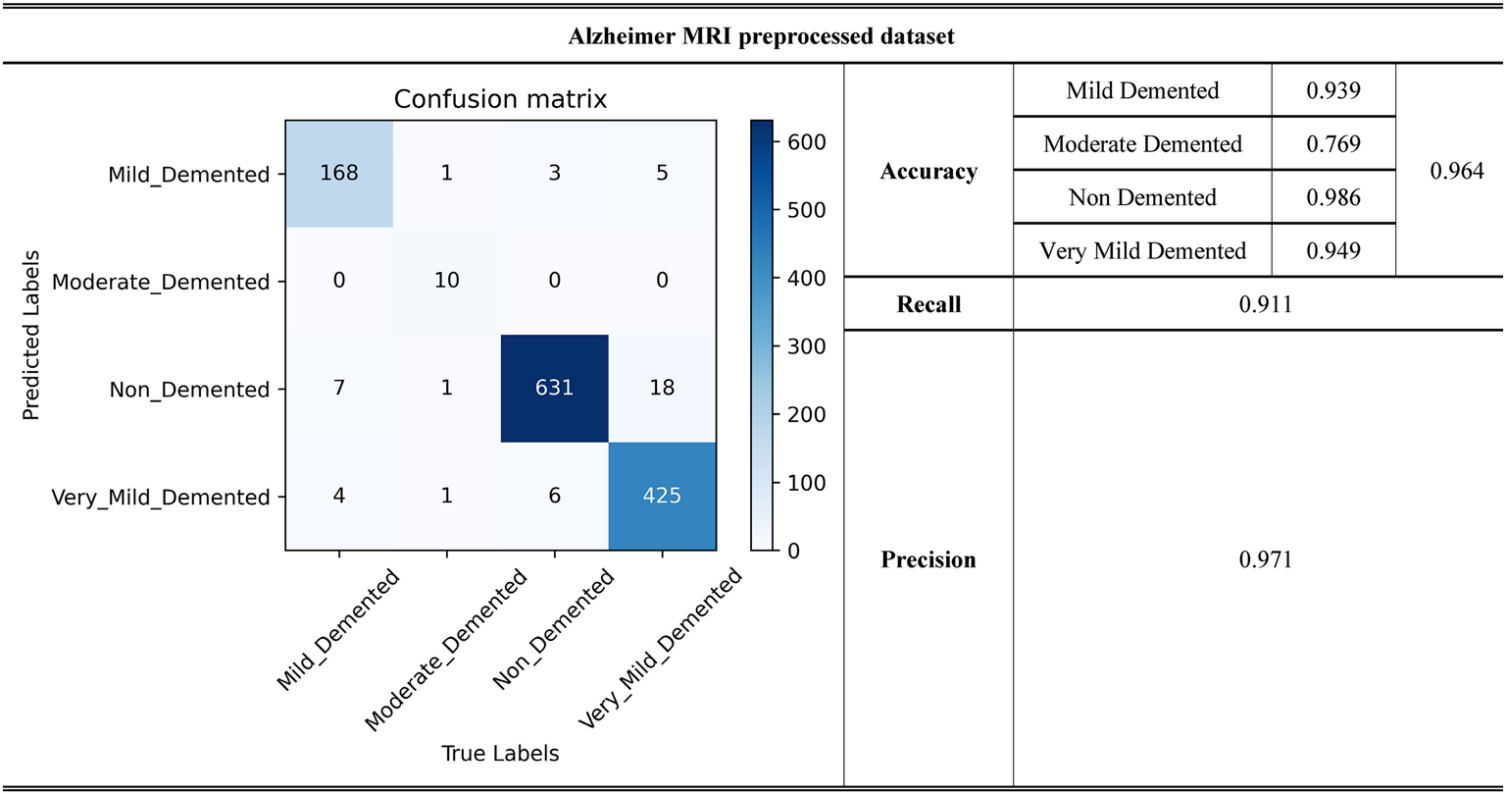
Prediction results of ResMini for the Alzheimer MRI preprocessed dataset.

Figure 4 presents the performance of ResMini on the Alzheimer MRI preprocessed dataset. The accuracy of 96.4%, recall of 91.1% and precision of 97.1% are obtained by ResMini, respectively. It demonstrates that ResMini behaves the robust performance and generalization capability when it is applied to different dataset. It should be noted that there is a performance disparity of ResMini across different classes for Alzheimer MRI preprocessed dataset, where Class Moderate Demented exhibits an average accuracy decrease of 18.9% compared to that of other Classes. This is due to an imbalance in sample sizes, where the sample number of Class Moderate Demented is at least less than one order compared with other Classes.

## Discussion

Aging is a gradual, multifactorial and time-dependent process, which characterized by functional loss, physiological and psychological damage when age increases. During aging, the human brain structure undergoes changes, including brain atrophy, thinning of the cortex, and decreased white matter connectivity. These age-associated alterations in brain morphology and function are implicated in the decline of cognitive faculties such as memory and processing speed, alongside an augmented susceptibility to neurodegenerative conditions like Alzheimer's disease. Consequently, the prediction of brain aging holds promise for the early detection of age-related neuropathological changes.

Magnetic Resonance Imaging (MRI) serves as a robust modality for delineating structural and morphological alterations within the brain attributable to neurodegenerative disorders, particularly affecting regions implicated in memory and cognition, such as the hippocampus and temporal cortex. Leveraging high-resolution structural MRI images, deep learning methodologies enable direct acquisition of salient features, thereby automating internal representation refinement and feature extraction processes, thereby enhancing efficacy in brain age prediction. In this paper, we developed a ResNet mini architecture that can learn the features and achieve better classification results in the task of brain age prediction from MRI images. Particularly, the proposed network can reduce the parameter number as much as possible under the premise of ensuring high accuracy and saving computing resources, allowing facilitated operation in the medical equipment. It is noteworthy that the ResMini has demonstrated remarkable performance across two different datasets by significantly reducing execution time and decreasing the parameter number. In addition, the ResMini has the ability to maintain stable and remarkable performance in dealing with imbalanced data distributions for the considered two datasets, allowing it to effectively handle the different sample numbers across different classes. The proposed ResMini architecture might be extended to solve the classification tasks for other similar neural images.

There are some limitations to this study. The medical diagnosis is a complex task, which needs to comprehensively consider the patient's medical history, clinical symptoms, physical examination, imaging results and other information. To serve accurate pathological diagnosis in solving complex task, it is necessary to construct clinical datasets in the future to train and validate the model.

## Materials and methods

### Datasets

In this study, the brain age dataset (OpenNeuro #ds000228) that contains functional magnetic resonance imaging (fMRI) recordings from a sample of adults and children watching a Pixar short film^23^ is first used to evaluate the performance of considered networks in this experiment. This dataset that obtained from 155 study participants can be available at https://openneuro.org/datasets/ds000228/versions/1.1.0, where the participants comprised 33 adults ranging from ages 18 to 39 (M_age_ = 24.8, SD_age_ = 5.3; 20 female) and 122 children (3–12 years old; M_age_ = 6.7, SD_age_ = 2.3; 64 female).

**Figure 5:**
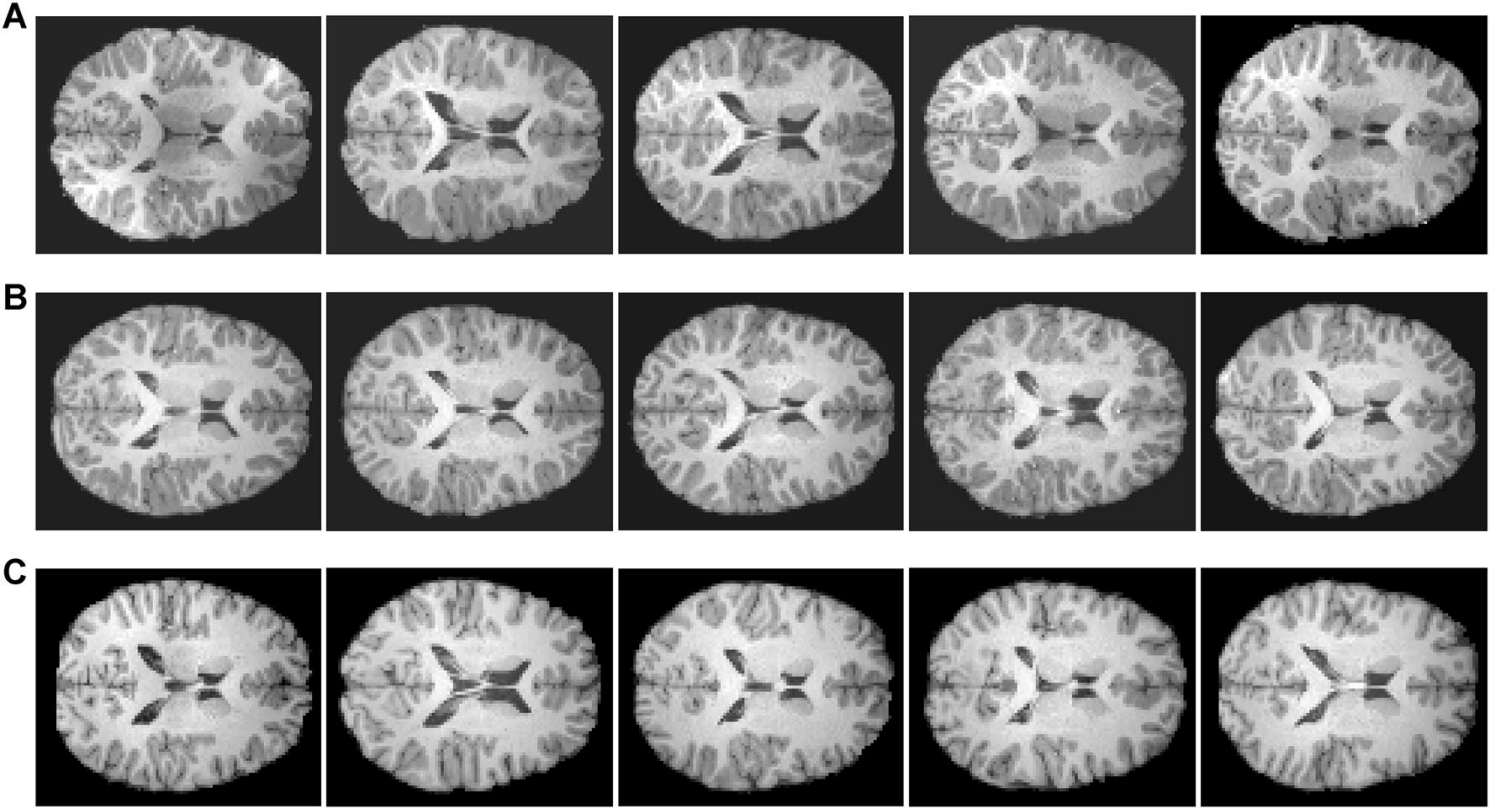
Example of 2D axial middle layer images in three age group categories of the brain age dataset (OpenNeuro #ds000228). Rows (**A**), (**B**) and (**C**) are randomly selected samples of 3–5 years old, 7–12 years old and adults, respectively.

Herein, we use two dimensional (2D) axial middle layer images of the brain age dataset. There are three age group categories: 3–5 years old, 7–12 years old and adults, as shown in Fig. 5. The 2D axial middle slice images are widely used in EEG MRI diagnosis, which can clearly show brain anatomy. By selecting an intermediate slice, it is possible to cover key areas of the brain, such as the cerebellum, cerebral hemispheres and brainstem, which helps doctors quickly understand the patient's brain anatomy and its changes. In terms of lesion detection and evaluation, doctors can look for pathological changes such as signals, tumors, and infarct areas from the middle section, and evaluate the relationship between their shape, size, location, and surrounding anatomical structures.

**Figure 6:**
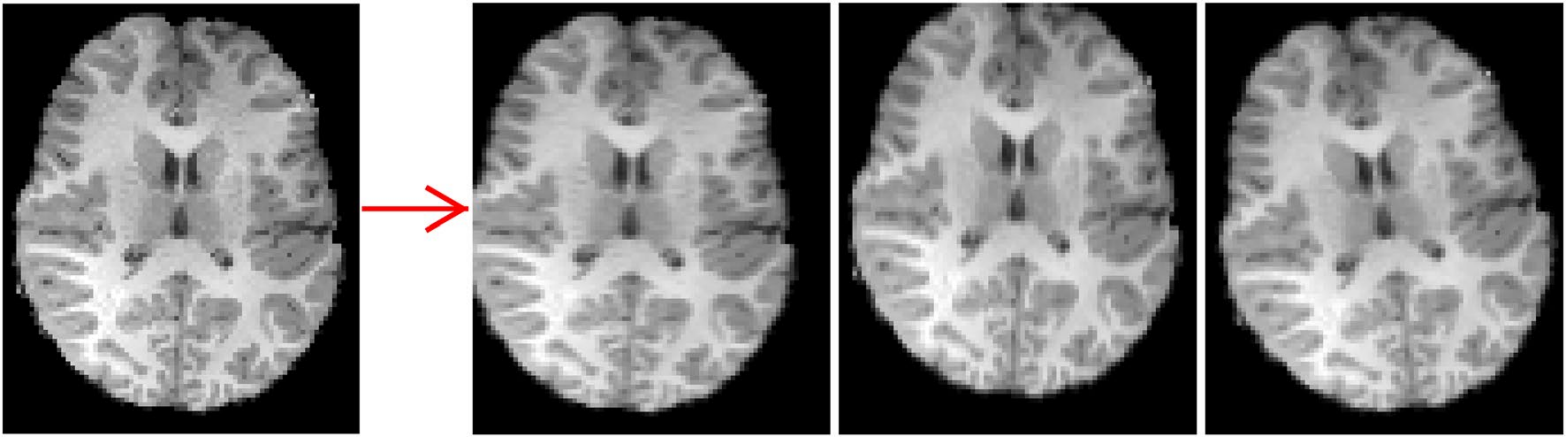
Image augmentation for the brain slice image of the brain age dataset (OpenNeuro #ds000228).

The brain age dataset contains a total of 155 images, each with a pixel size of 95 × 79, which is insufficient to support deep learning training. To mitigate overfitting and prevent the network from memorizing precise details of the training images, we conducted preprocessing on the data. This involved introducing horizontal and vertical offsets to the images, and performing horizontal axis flipping as shown in Fig. 6. This approach aimed to augment the data set, providing a richer and more balanced set of images. Subsequently, a total of 310 samples are obtained, including adults of 66 samples, 3–5 years old of 130 samples, and 7–12 years old of 114 samples.

To validate the robustness and generalization capabilities of ResMini, the Alzheimer MRI dataset (https://www.kaggle.com/datasets/sachinkumar413/alzheimer-mri-dataset) is included in our experiment. The Alzheimer MRI dataset comprises four classes of images (Mild Demented, Moderate Demented, Non Demented, and Very Mild Demented) with sample sizes of 896, 64, 3200, and 2240, respectively.

In practice, each of the two datasets was partitioned into a training set and a test set at an 8:2 ratio. Additionally, within the training set, a further division was made into a training set and a validation set at an 8:2 ratio.

### ResMini block

**Figure 7:**
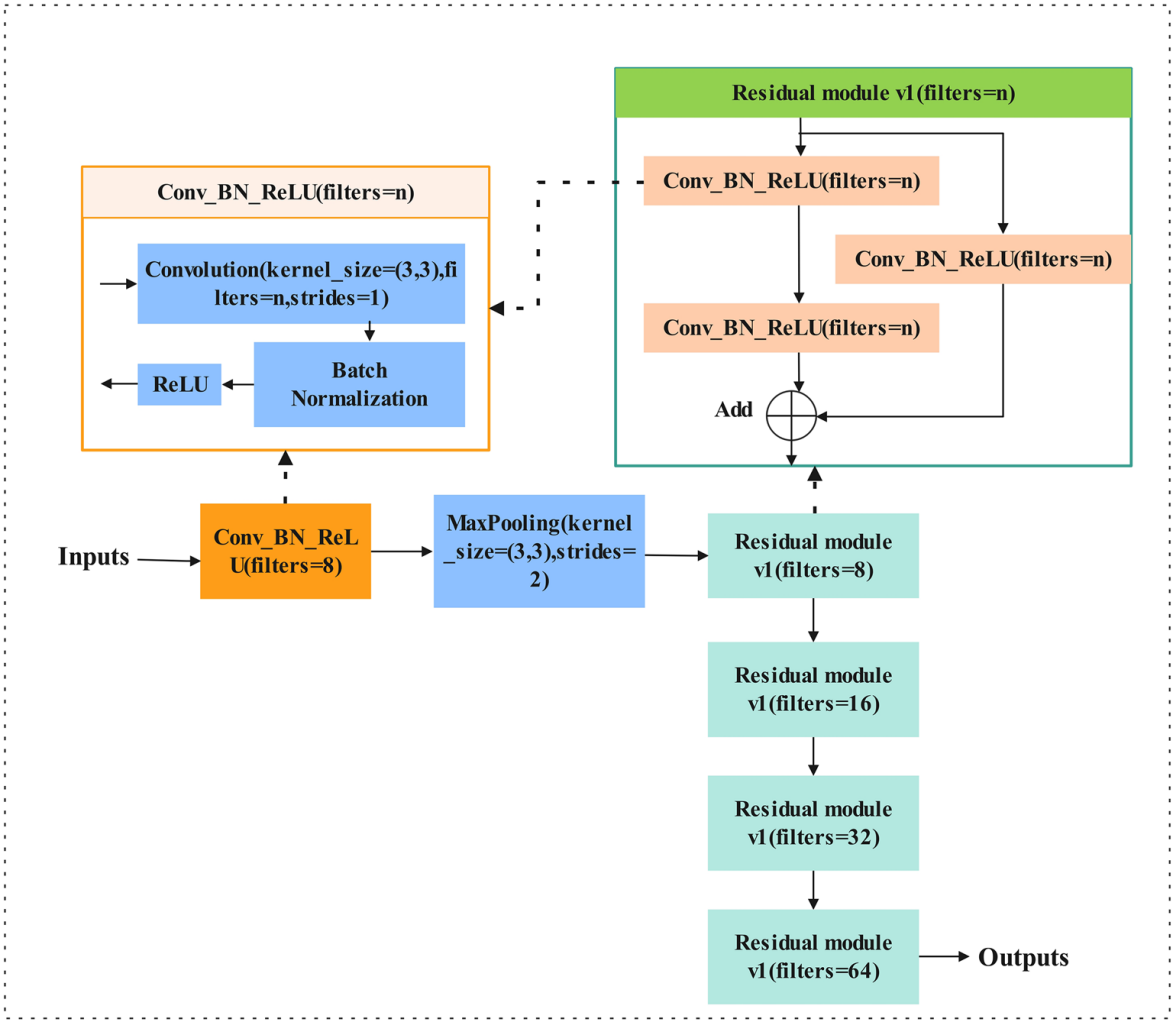
ResMini architecture.

In this section, we introduce the ResMini architecture that modified by the ResNet^20^. ResNet was originally designed to handle large data sets (such as ImageNet) and complex tasks (such as image classification, object detection, etc.), often at deep depths, such as ResNet-50, ResNet-101, etc. These deep networks perform well on large data sets due to their large number of parameters and complex structure, which allows them to capture high-level features and patterns in the data. However, the brain age dataset has a total of 310 samples after data enhancement, which is only 0.002% of ImageNet. Nevertheless, we tried the brain age dataset and found that 10-layer ResNet worked well. The depth of ResMini is only 10 layers, which can significantly reduce network parameters while retaining the accuracy and reducing training time. Figure 7 depicts the ResMini architecture when we apply it to solve our study task. The details of the ResNet mini are briefly summarized as follows: it consists of a basic block called Conv_BN_ReLU^24^, followed by a maximum pooling layer, four residual modules, an average pooling layer, a fully connected layer, and finally a softmax.

In the Cov_BN_ReLU block, batch normalization (BN)^25^ is added between a convolutional layer with a kernel size of 3 × 3 and a step size of 1 and a padding of 3 pixels and the largest pooling layer with a core size of 3 × 3 and a stride of 2 and a padding of 1 pixel. This integration significantly enhances model training efficiency and expedites convergence. The Residuals module comprises three Conv_BN_ReLU blocks, as depicted in the upper right corner of Fig. 7. As the network deepens, the number of filters in the residual module convolutional layer also doubles. Subsequently, the final residual module is linked to an average pooling layer, followed by a fully connected layer comprising three neurons for the brain age dataset, and four neurons for the Alzheimer's MRI preprocessed dataset. Ultimately, the network yields the final classification result through the softmax function at its terminus.

### Experimental platform

In this study, the multi-channel fusion model was implemented using Python (version 3.9.7), using the open-source deep learning framework Tensorflow (version 2.5.0), the experimental platform was a Lenovo server, the physical memory was 32G, the CPU model was Intel(R) Xeon(R) Silver 4210R CPU @ 2.40 GHz, and the graphics card model was NVIDIA GeForce RTX 3080 Ti (12G) with Ubuntu 18.04.6 LTS installed on the physical machine.

### Parameter setting

We chose the ADAM optimization algorithm. ADAM is a variation of the gradient descent algorithm, but the learning rate of the parameters in each iteration has a certain range, and the learning rate (step size) will not become large because the gradient is large, and the value of the parameters is relatively stable. We set the learning rate to 0.00002, the number of input network samples at a time is set to 24, and the number of training rounds is set to 200.

## Data Availability

The dataset used and/or analysed during the current study is available from OpenNEURO platform (https://openneuro.org/datasets/ds000228/versions/1.1.0) and the Kaggle platform (https://www.kaggle.com/datasets/sachinkumar413/alzheimer-mri-dataset).
